# Supplementary material for: Predicting Humoral Alloimmunity from Differences in Donor and Recipient HLA Surface Electrostatic Potential
Source: J Immunol. 2018 Nov 14;201(12):3780–92. doi: 10.4049/jimmunol.1800683 (PMC6287104; doi:10.4049/jimmunol.1800683)
Supplement: Data Supplement [file JI_1800683.zip › JI_1800683_Supplemental_Material_1.pdf]

Supplemental Information

Supplementary Table S1. Amino acid sequence polymorphism and surface electrostatic potential differences among HLA class I and class II alleles

Locus	Amino acid sequence polymorphism	Electrostatic similarity distance
**HLA-A**	5.32 (3.72 - 6.65)	0.355 (0.293 - 0.456)
**HLA-B**	5.05 (3.99 - 5.85)	0.313 (0.228 - 0.382)
**HLA-C**	3.72 (2.66 - 4.52)	0.349 (0.276 - 0.399)
**HLA-DR**	4.18 (2.61 - 5.22)	0.276 (0.214 - 0.332)
**HLA-DQ**	8.36 (4.58 - 11.59)	0.407 (0.322 - 0.452)
**HLA-DP**	3.83 (2.73 - 5.19)	0.261 (0.190 - 0.319)

Pair-wise, all-versus-all comparisons of amino acid sequence and of surface electrostatic potential were performed between common (frequency >1%) HLA alleles within individual HLA class I and class II loci. The Table shows the median (interquartile range) values for HLA allele comparisons within individual loci.

Supplementary Figure S1. Relationship between sequence polymorphism and surface electrostatic potential differences among HLA class I and class II molecules.

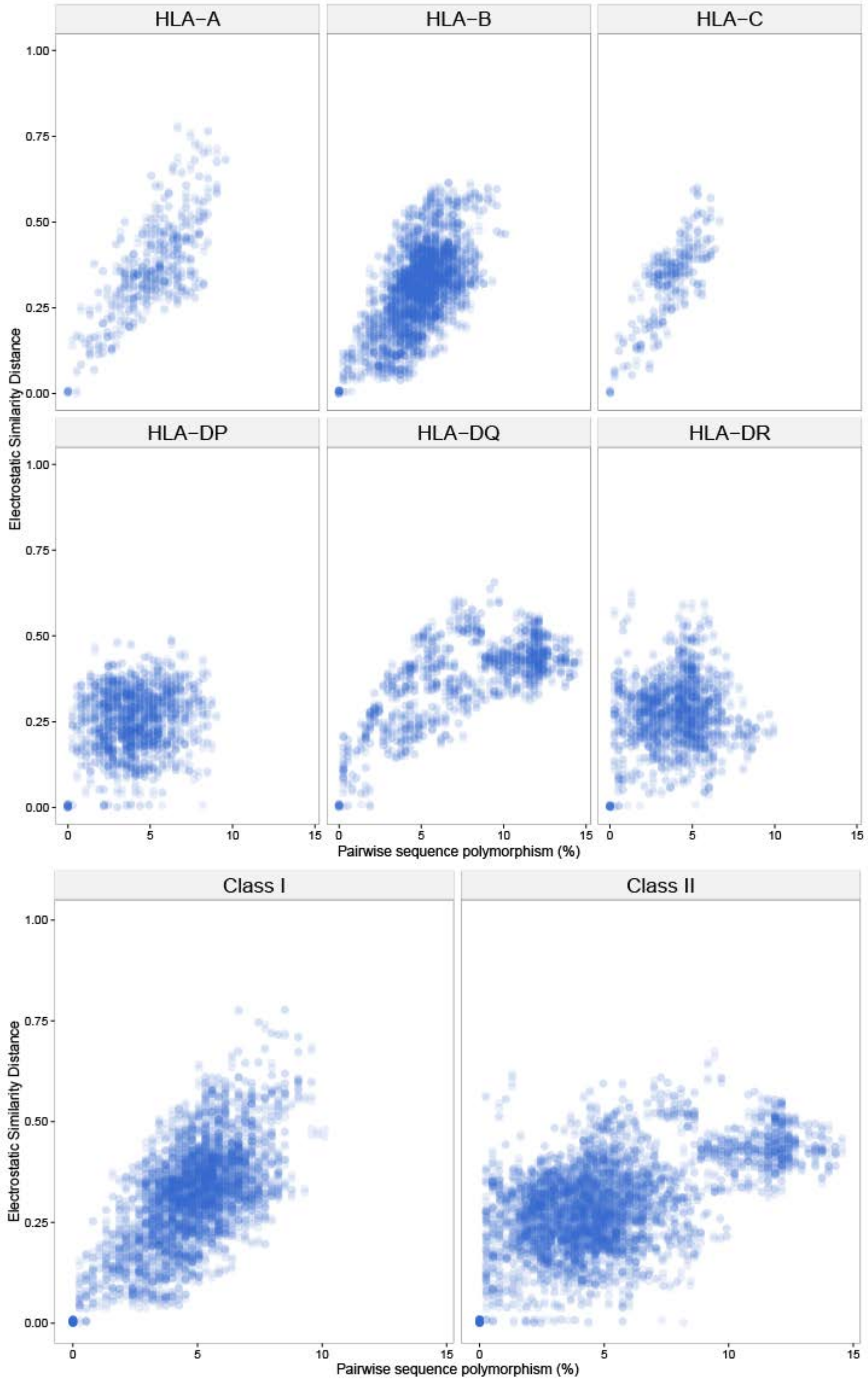

Pair-wise, all-versus-all comparisons of amino acid sequence and of surface electrostatic potential were performed between common (frequency >1%) HLA alleles within individual HLA class I and class II loci. The figure depicts the relationship between amino acid polymorphism and electrostatic potential differences (electrostatic similarity distance, ESD) for compared HLA-A, -B, -C, -DR, -DQ, and -DP alleles. The correlation (R^2) between amino acid sequence polymorphism and ESD was 0.608 for HLA-A, 0.412 for HLA-B, 0.691 for HLA-C, 0.018 for HLA-DR, 0.507 for HLA-DQ, and 0.053 for HLA-DP (0.439 and 0.317 for HLA class I and class II respectively).

Supplementary Figure S2. Electrostatic similarity distance among HLA alleles within individual HLA-A, -B, -C, -DR, -DQ and -DP loci.

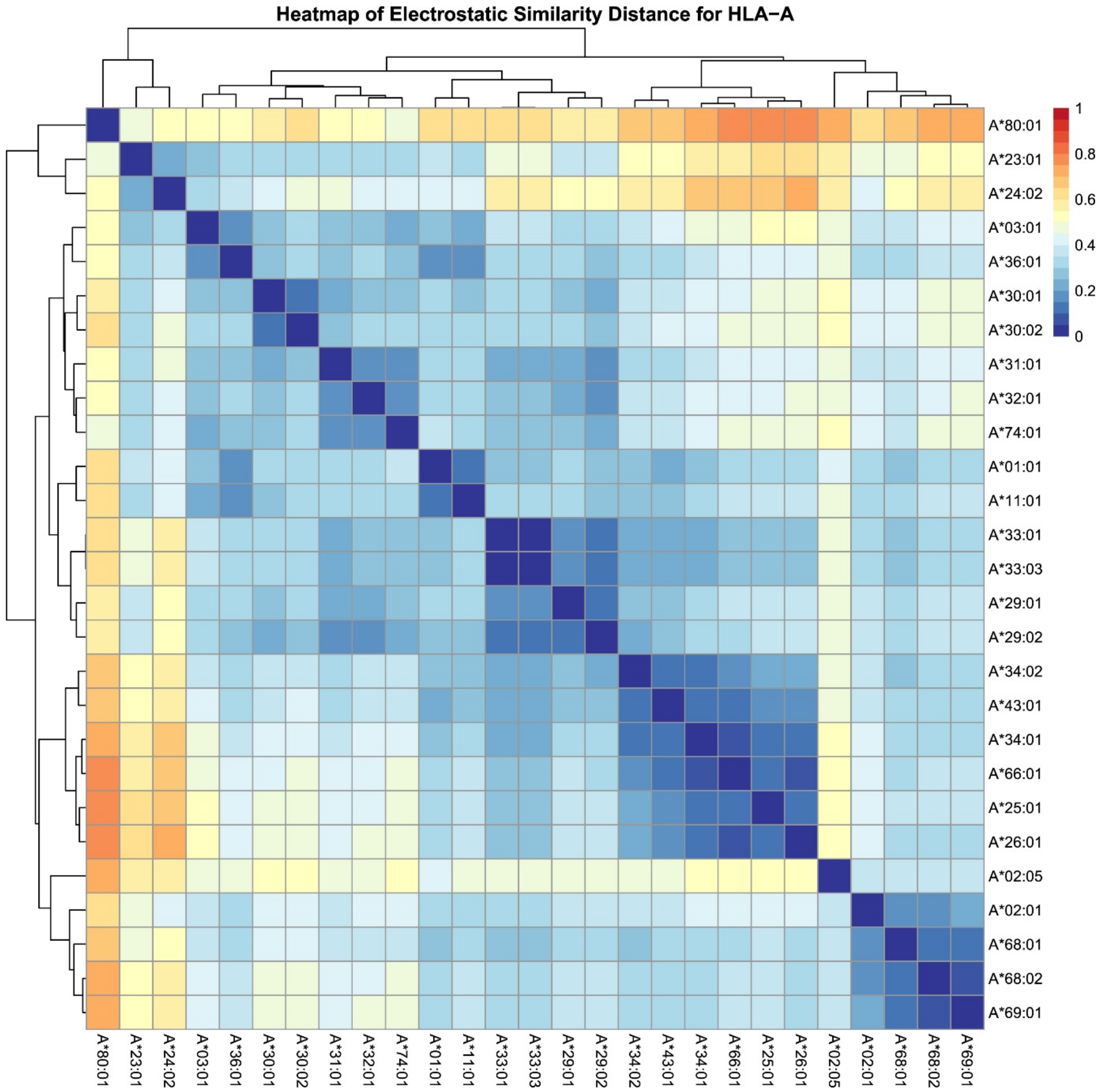

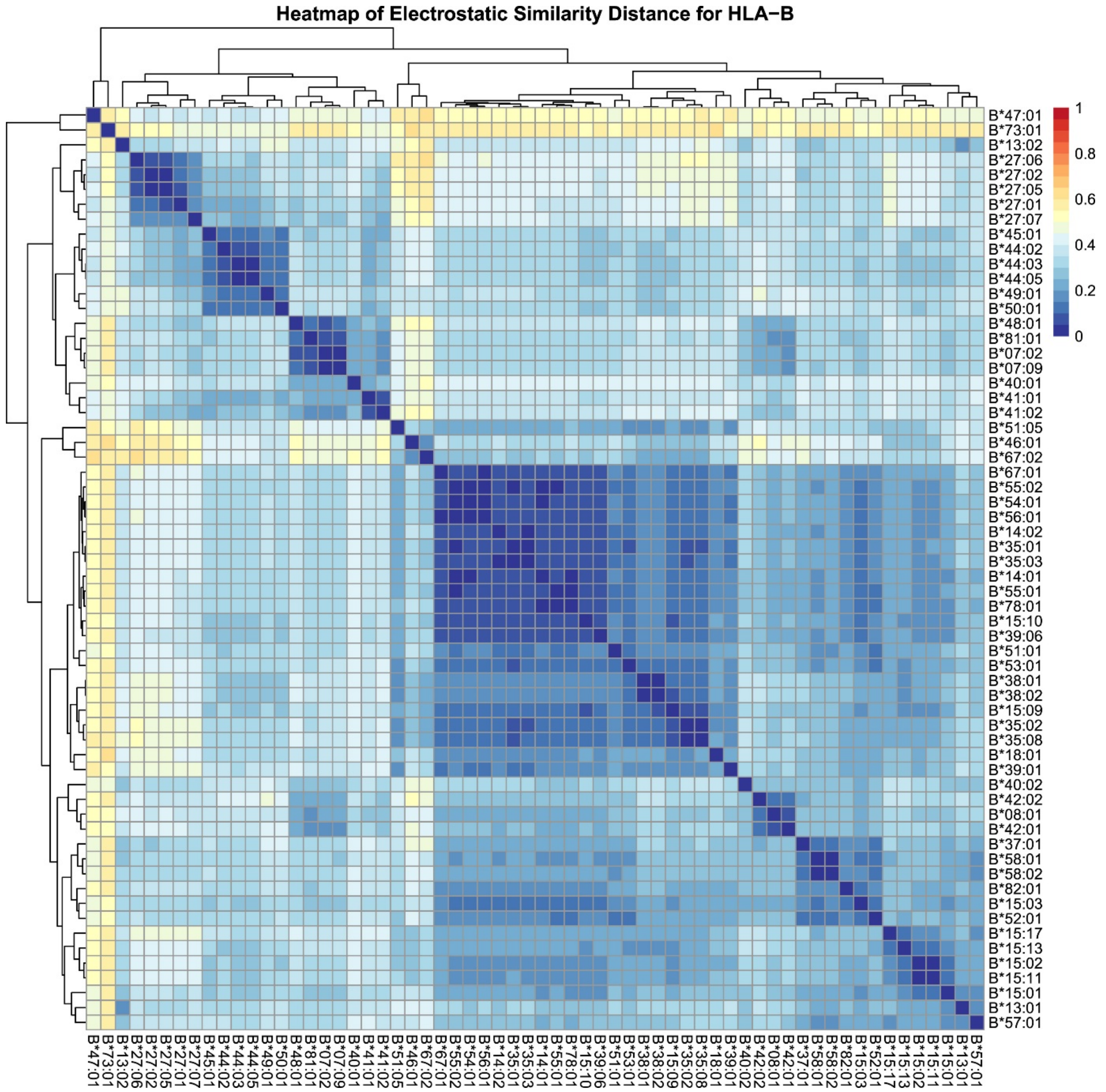
Heatmap of Electrostatic Similarity Distance for HLA−B
1
0.8
0.6
0.4
0.2
0
B*47:01
B*73:01
B*13:02
B*27:06
B*27:02
B*27:05
B*27:01
B*27:07
B*45:01
B*44:02
B*44:03
B*44:05
B*49:01
B*50:01
B*48:01
B*81:01
B*07:02
B*07:09
B*40:01
B*41:01
B*41:02
B*51:05
B*46:01
B*67:02
B*67:01
B*55:02
B*54:01
B*56:01
B*14:02
B*35:01
B*35:03
B*14:01
B*55:01
B*78:01
B*15:10
B*39:06
B*51:01
B*53:01
B*38:01
B*38:02
B*15:09
B*35:02
B*35:08
B*18:01
B*39:01
B*40:02
B*42:02
B*08:01
B*42:01
B*37:01
B*58:01
B*58:02
B*82:01
B*15:03
B*52:01
B*15:17
B*15:13
B*15:02
B*15:11
B*15:01
B*13:01
B*57:01

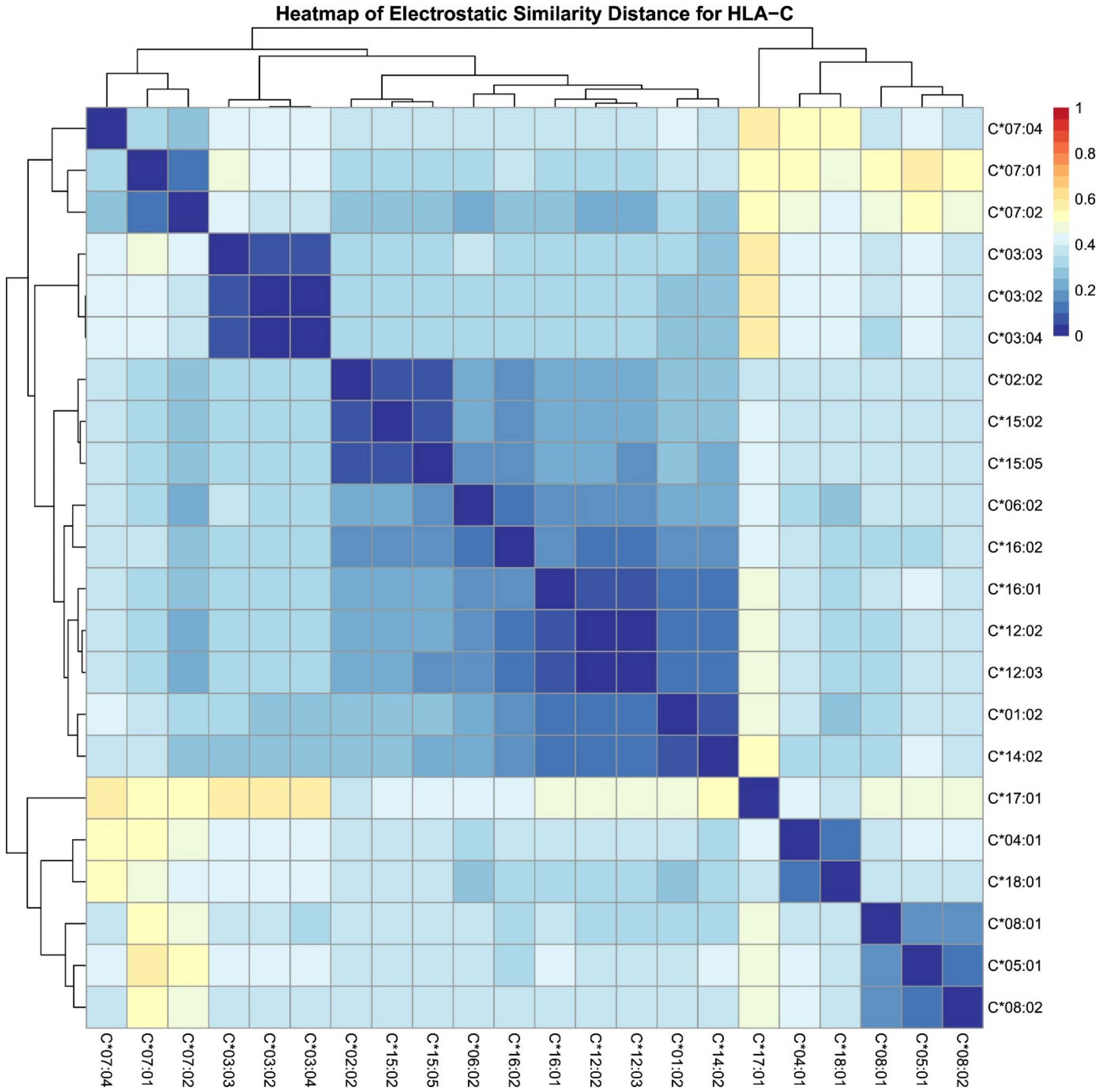

Heatmap of Electrostatic Similarity Distance for HLA−C
1
0.8
0.6
0.4
0.2
0
C*07:04
C*07:01
C*07:02
C*03:03
C*03:02
C*03:04
C*02:02
C*15:02
C*15:05
C*06:02
C*16:02
C*16:01
C*12:02
C*12:03
C*01:02
C*14:02
C*17:01
C*04:01
C*18:01
C*08:01
C*05:01
C*08:02

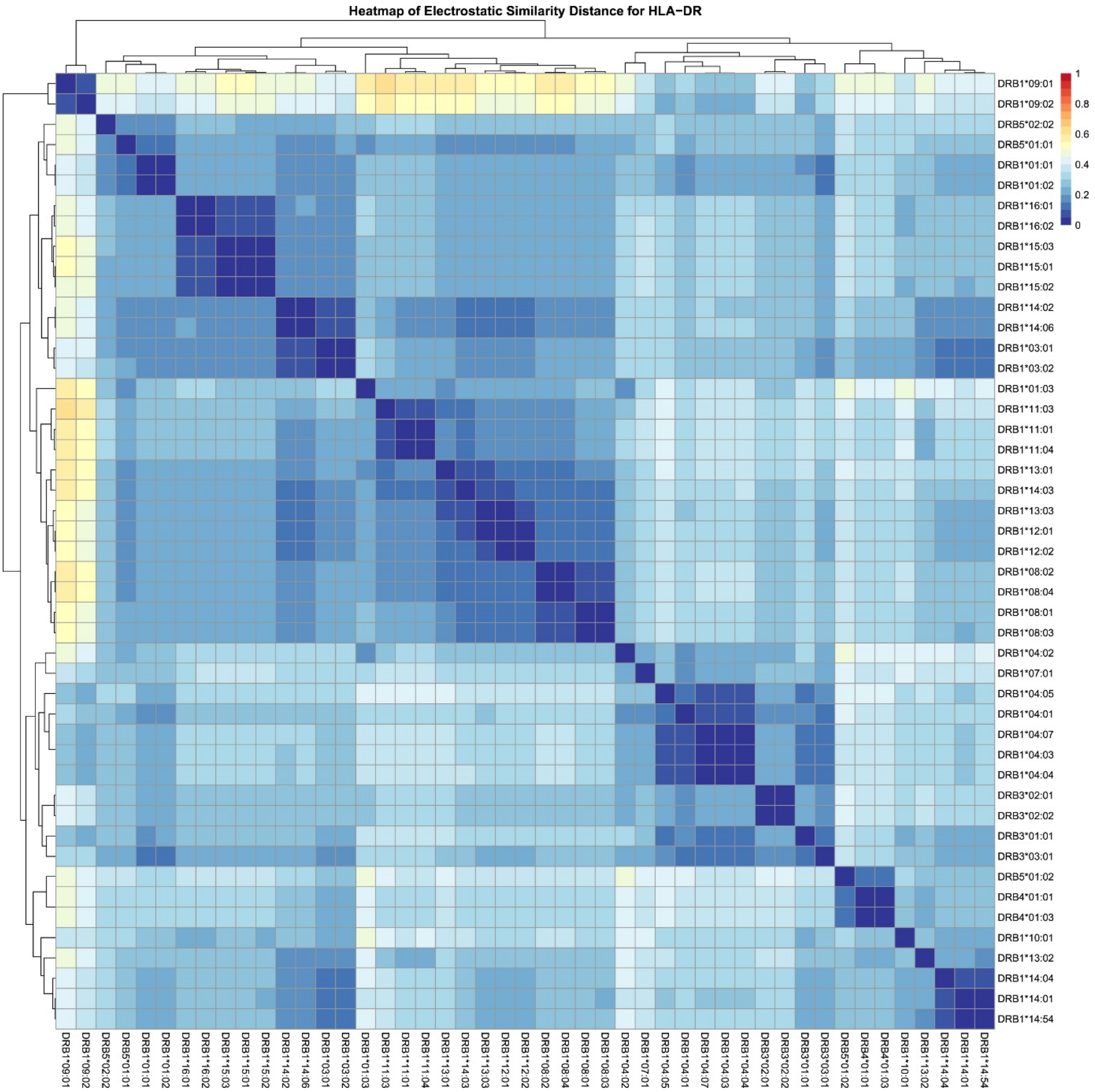
Heatmap of Electrostatic Similarity Distance for HLA−DR
1
0.8
0.6
0.4
0.2
0
DRB1*09:01
DRB1*09:02
DRB5*02:02
DRB5*01:01
DRB1*01:01
DRB1*01:02
DRB1*16:01
DRB1*16:02
DRB1*15:03
DRB1*15:01
DRB1*15:02
DRB1*14:02
DRB1*14:06
DRB1*03:01
DRB1*03:02
DRB1*01:03
DRB1*11:03
DRB1*11:01
DRB1*11:04
DRB1*13:01
DRB1*14:03
DRB1*13:03
DRB1*12:01
DRB1*12:02
DRB1*08:02
DRB1*08:04
DRB1*08:01
DRB1*08:03
DRB1*04:02
DRB1*07:01
DRB1*04:05
DRB1*04:01
DRB1*04:07
DRB1*04:03
DRB1*04:04
DRB3*02:01
DRB3*02:02
DRB3*01:01
DRB3*03:01
DRB5*01:02
DRB4*01:01
DRB4*01:03
DRB1*10:01
DRB1*13:02
DRB1*14:04
DRB1*14:01
DRB1*14:54

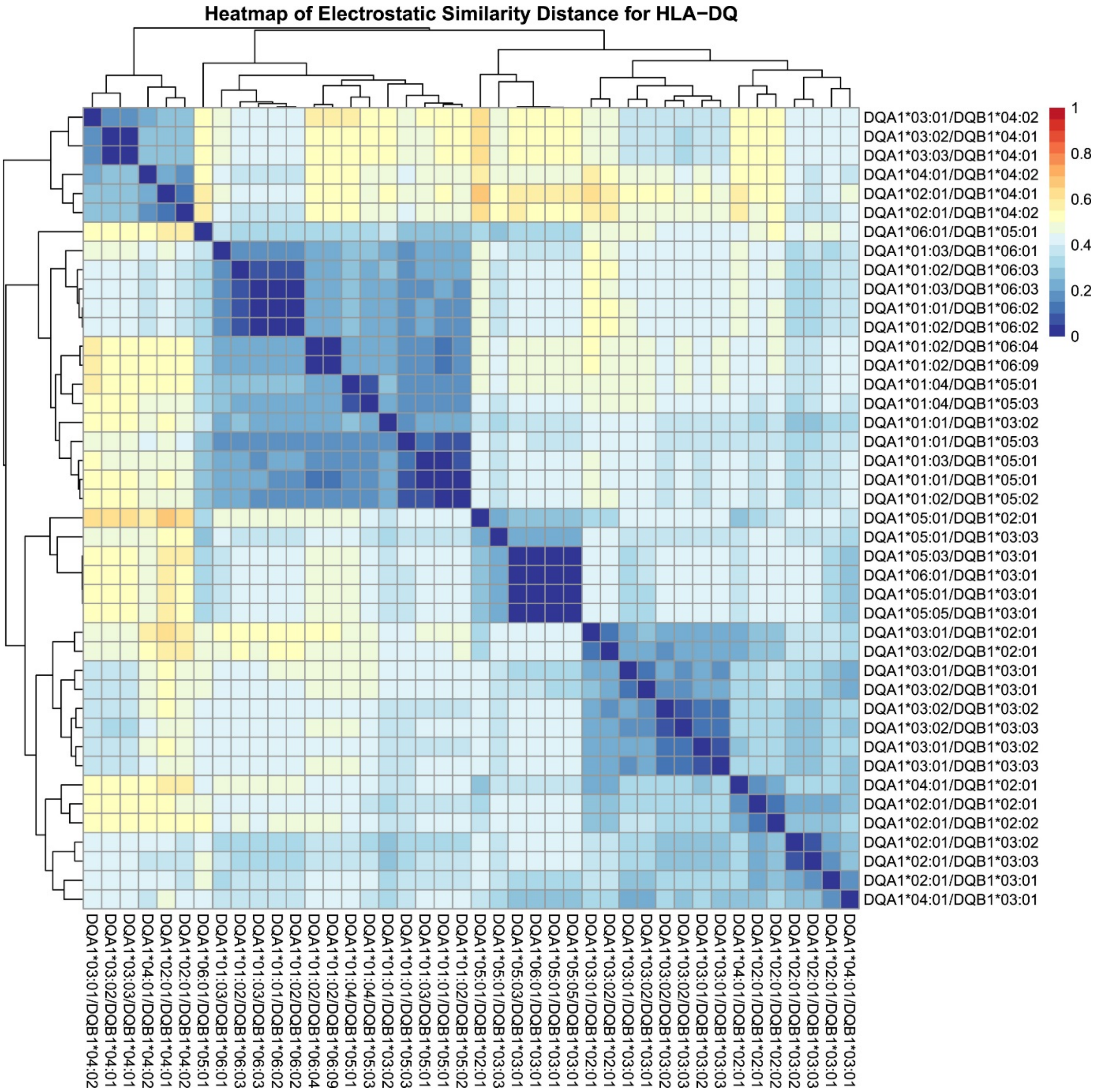
Heatmap of Electrostatic Similarity Distance for HLA−DQ
DQA1*03:01/DQB1*04:02
DQA1*03:02/DQB1*04:01
DQA1*03:03/DQB1*04:01
DQA1*04:01/DQB1*04:02
DQA1*02:01/DQB1*04:01
DQA1*02:01/DQB1*04:02
DQA1*06:01/DQB1*05:01
DQA1*01:03/DQB1*06:01
DQA1*01:02/DQB1*06:03
DQA1*01:03/DQB1*06:03
DQA1*01:01/DQB1*06:02
DQA1*01:02/DQB1*06:02
DQA1*01:02/DQB1*06:04
DQA1*01:02/DQB1*06:09
DQA1*01:04/DQB1*05:01
DQA1*01:04/DQB1*05:03
DQA1*01:01/DQB1*03:02
DQA1*01:01/DQB1*05:03
DQA1*01:03/DQB1*05:01
DQA1*01:01/DQB1*05:01
DQA1*01:02/DQB1*05:02
DQA1*05:01/DQB1*02:01
DQA1*05:01/DQB1*03:03
DQA1*05:03/DQB1*03:01
DQA1*06:01/DQB1*03:01
DQA1*05:01/DQB1*03:01
DQA1*05:05/DQB1*03:01
DQA1*03:01/DQB1*02:01
DQA1*03:02/DQB1*02:01
DQA1*03:01/DQB1*03:01
DQA1*03:02/DQB1*03:01
DQA1*03:02/DQB1*03:02
DQA1*03:02/DQB1*03:03
DQA1*03:01/DQB1*03:02
DQA1*03:01/DQB1*03:03
DQA1*04:01/DQB1*02:01
DQA1*02:01/DQB1*02:01
DQA1*02:01/DQB1*02:02
DQA1*02:01/DQB1*03:02
DQA1*02:01/DQB1*03:03
DQA1*02:01/DQB1*03:01
DQA1*04:01/DQB1*03:01
1
0.8
0.6
0.4
0.2
0

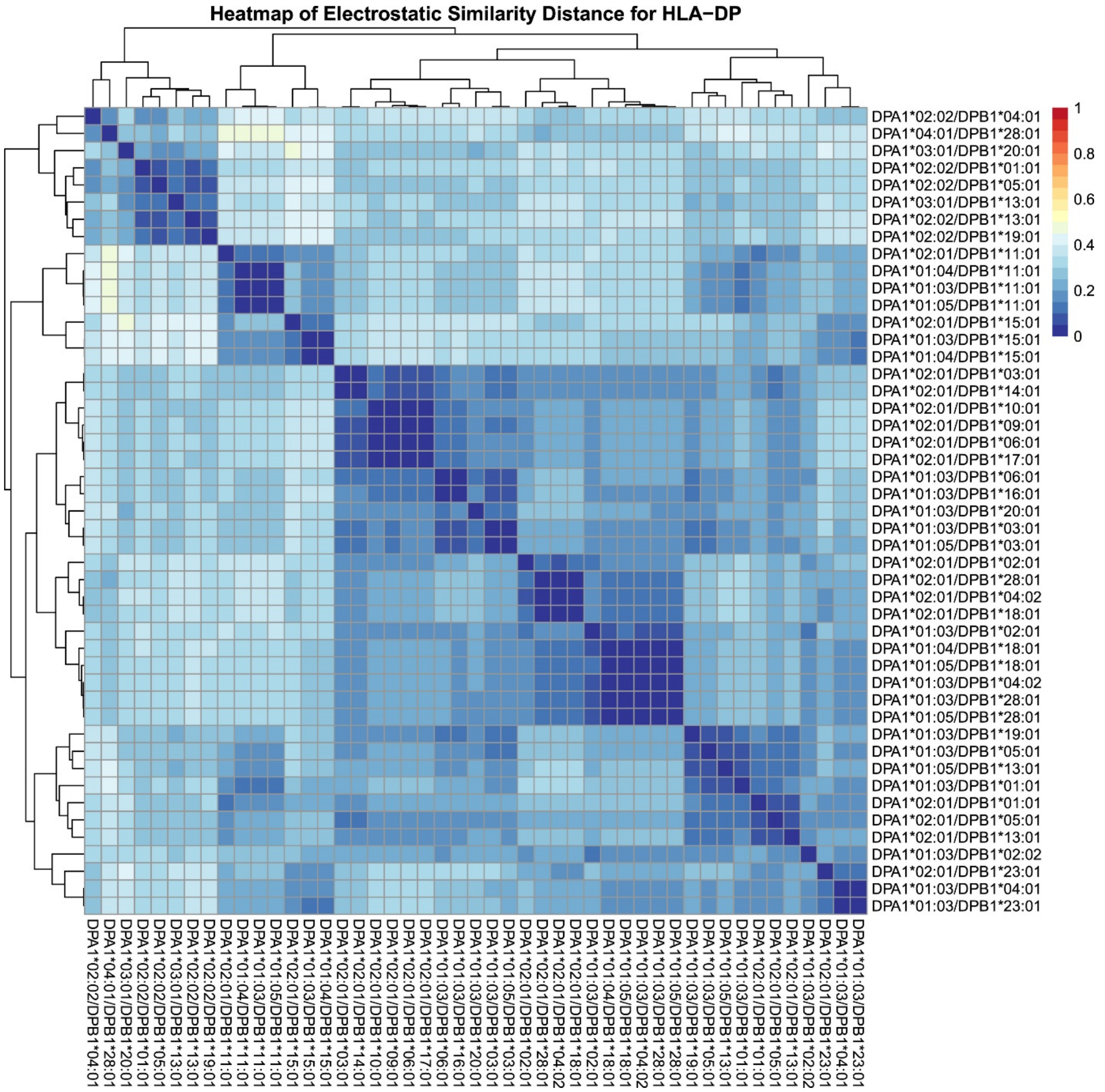

For HLA alleles within a locus, electrostatic potential comparisons were made in a pair-wise, all-versus-all fashion. The electrostatic similarity distances (ESD) generated by the comparisons were compiled as a distance matrix that is displayed as a symmetrical heatmap and as a dendrogram with allele re-ordering such that electrostatically similar alleles cluster together. Blue colour denotes electrostatic similarity and red colour depicts electrostatic dissimilarity (according to the ESD scale shown).

Supplementary Figure S3. Frequency of donor HLA-C mismatches in the lymphocyte immunotherapy patient cohort according to their Electrostatic Mismatch Score (EMS-3D).

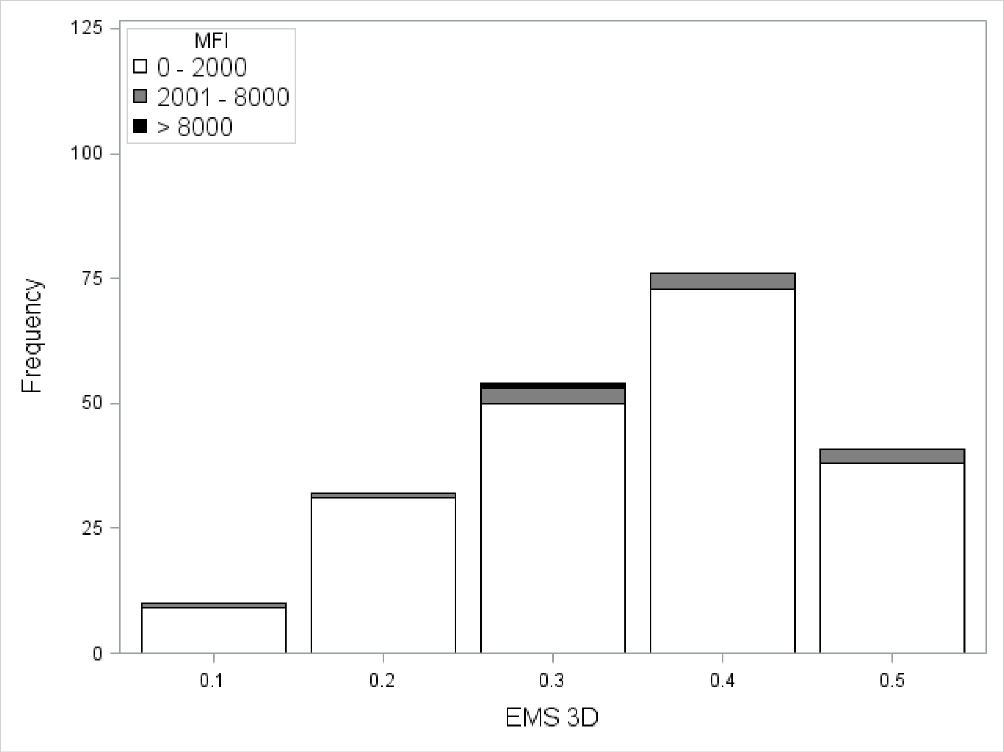

The figure depicts the frequency of donor-recipient HLA-C mismatches according to their EMS-3D. The frequency of donor-specific alloantibody development against HLA-C mismatches is also shown, grouped according to their mean fluorescence intensity (MFI) on the Luminex single antigen bead assay.
